# Supplementary material for: Divergent combinations of cis-regulatory elements control the evolution of phenotypic plasticity
Source: PLoS Biol. 2023 Aug 17;21(8):e3002270. doi: 10.1371/journal.pbio.3002270 (PMC10464979; doi:10.1371/journal.pbio.3002270)
Supplement: S1 Text — (DOCX) [file pbio.3002270.s001.docx]

**Divergent combinations of *cis*-regulatory elements control the evolution of phenotypic plasticity**

Mohannad Dardiry, Gabi Eberhard, Hanh Witte, Christian Rödelsperger, James W. Lightfoot & Ralf J. Sommer

Supplementary Materials

Materials and Methods

**Other Supplementary Materials for this manuscript include the following:**

Figs. S1 to S9

Tables S1 to S5

Data S1 to S9

Materials and Methods

**Nematode culture maintenance**

*P. pacificus* natural isolates were reared on the standard laboratory *E. coli* strain OP50 as previously described in (1). Original strain isolation and genomic characterization are found in (2). All strains studied in this analysis belong to *P. pacificus* clade B, one of four major clades of this species. Clade B strains are restricted to high altitude localities on La Réunion Island as previously described in (2).

**Mouth-form phenotyping**

The mouth form of individual adults was characterized based on the width of the mouth, and the shape of the dorsal tooth as previously described in (3). Screening was done on a ZEISS SteREO Discovery.V20 microscope, PlanApo S 1.5x objective with eyepiece PL 10x/23 Br.foc. Three to five replicates were done for each experiment with a total of 150-250 animals. All mouth-form screening was done at 20° C on a 300µl lawn of OP50 bacteria.

**Generation of recombinant inbred lines (RIL) and mouth-form screening**

To produce RILs, we crossed individuals of the two parental lines. Specifically, we used a reciprocal setup with five adult males from RSA076 and two old hermaphrodites from RSC011 that do not produce any self-sperm anymore; and vice versa. After two days, parents were removed and 10 F1 animals were singled out. In total, 160 hermaphroditic F1 animals were isolated as J4 juveniles to avoid any possible mating with F1 males. After F1 animals had laid eggs, they were lysed to extract DNA. We performed single worm PCRs as previously described (19) to confirm the hybrid nature of the F1 animals using a SNP between the two parental lines RSA076 and RSC011; Cytosine (C) in RSA076, while Guanine (G) in RSC011 at genomic position (Chr.1 12,217,846). The following primers were used for amplification.

| Primer name | Primer sequence 5’------>3’ | Primer function |
| --- | --- | --- |
| MD15650 | TCTCGCTTGTGCTATCGGA | Forward PCR primer |
| MD15652 | TGTTGCAGCTGGCTAACTCAC | Reverse PCR primer |
| MD15653 | CGAGTTACCTCCTCA | Sequencing Primer |

After heterozygosity was confirmed, one J4 animal from each of 160 lines were transferred to a new plate. For at least 12 generations, single worms were transferred from each line. Supposedly at F8, homozygous RILs with mosaic genetic background were generated. During the generation of the F12 RILs, we lost a total of 20 lines. From the remaining 140 lines, we have randomly chosen 55 lines and screened mouth-form scores for five successive generations (F12 - F16).

For the between clade RIL experiment, we followed the same setup mentioned above. We used the reference strain PS312 from California as representative for clade A and RSB020 as a representative for clade C. In total, 240 hermaphroditic F1 animals were isolated and phenotypic scoring was performed for 120 lines. From which 94 lines were selected to score the phenotype for two to three successive generations. Finally, these 94 lines were used for downstream analysis. To confirm the production of hybrids in this setup, we used a SNP at the genomic position (ChrV. 10,737,477) between the two parents as marker (PS312: Cytosine, RSB020 Adenine). The following primers were used for amplification.

| Primer name | Primer sequence 5’------>3’ | Primer function |
| --- | --- | --- |
| MD15288 | TAGGATACAATGGGCTTCCCTC | Forward PCR primer |
| MD15289 | AGAATACTGTGTTTCCTACTCCGC | Reverse PCR primer |
| MD15290 | AATGGGCTTCCCTCTAC | Sequencing Primer |

**Whole-genome and RAD-seq sequencing**

For the within clade analysis (RSA076 and RSC011), we performed whole-genome sequencing on 55 lines. We used 40 ml 0.9% NaCl solution to wash five fully grown plates into 50 ml falcon tubes. After two initial washing steps, worms were incubated overnight in 40 ml 0.9% NaCl with 40µl Ampicillin (50µg/ml) and 40µl Chloramphenicol (50µg/ml) at room temperature. Next day, worm pellets were transferred into 1.5 ml Eppendorf tubes and after centrifugation (140000 rpm for 1 min at 20 °C) worm pellets were frozen at -20°C. We conducted three freezing/thawing cycles using liquid nitrogen and the thermomixer at 37 °C. Subsequently, DNA was extracted using GeneElute Mammalian Genomic DNA Miniprep Kit (DNA column purification, Sigma G1N70) following the manufacturer's protocol using 200µl Lysis solution T (B6678) and 50µl of proteinase K (10 mg/ml) for digestion. Next generation DNA sequencing libraries (NGS) were prepared using the Nextera DNA Flex Library Prep Kit (illumina) following the manufacturer’s protocol. Libraries were paired-end sequenced using an inhouse HiSeq 3000 machine (Illumina). For the between clades analysis (PS312 and RSB020), we performed RAD-seq on 96 lines. RADseq was performed as described in (4). DNA was extracted using the epicentre MasterPure DNA Purification Kit following the manufacturer’s protocol (Biozym Scientific GmbH, MCD85201). To select fragments of 250-500 bp, we used bluepippin (Sage Science, Massachusetts, USA) following the manufacturer’s protocol. Libraries were sequenced using an inhouse HiSeq 3000 machine (Illumina). For both library preparation methodologies, DNA quality and quantity was validated with a Qubit 2.0 fluorometer (Life Technologies GmbH, Darmstadt, Germany) and fragment length confirmation was performed using Agilent Bioanalyzer (Agilent Technologies GmbH, Waldbronn, Germany).

**Identification of the candidate region (QTL mapping) and genomic variants**

For the cross between RSC011 and RSA076, raw sequence reads were aligned against the *P. pacificus* PS312 reference assembly (version El Paco) with the BWA aln and same programs (version 0.7.17-r1188) using default options (10,22). Informative marker positions between the two parental strains RSC011 and RSA076 were identified using a different variant calling approach (5). The classification of the differential variants into non-coding, nonsynonymous, synonymous substitutions was done as described previously (6). Alignments of RILs were genotyped at the informative marker positions using the differential variant calling approach (at least 5X coverage). For the RILs between PS312 and RSB020, raw RAD-seq data were aligned to the reference assembly by the BWA aln and sampe programs. Variant positions in the RSB020 genome were extracted from the data set from (7) and genotyped in the alignment file for each RIL using samtools mpileup (version 0.1.18-r982:295) and bcftools (version 0.1.17-dev-r973:277) programs as described in (7)*.* Only genotypes with a minimal quality score of 20 were retained. For markers with at least five instances of each of the parental genotypes, LOD scores were computed as the negative logarithm (base 10) of the P-value, as computed by Fisher's exact test. And a threshold corrected LOD score was set at 6 to signify the association between the genomic region and the phenotype.

**CRISPR/Cas-9 knockouts and swaps in the multi-gene locus**

We used available sequenced genomes of the clade B strain RSB0001 for variant calling, to identify candidates in the promoter region of *eud-1*. Genomic Sequence bed files were aligned to the annotated reference genome of RSB001 using the software IGV to identify candidates.

| **Primer name** | **Gene/regulatory element** | **Primer function** | **Primer sequence 5’--->3’** |
| --- | --- | --- | --- |
| MD019 | *nag-2 gene* | Forward primer | TCGGTGCATCTGGTAAGCT |
| MD022 | *nag-2 gene* | Reverse primer | TGTTATAATCCGACCGAATGC |
| MD024 | *nag-2 gene* | Sequencing primer | CATCTGGTAAGCTTGGTCT |
| MD007 | *eud-1 intron SNP* | Forward primer | CGCTAGTTGGTTCGCTCATA |
| MD009 | *eud-1 intron SNP* | Reverse primer | GTATGATGACGTTGGGATGC |
| MD013 | *eud-1 intron SNP* | Sequencing primer | TAGTTGGTTCGCTCATATC |
| MD014 | *eud-1* promoter region (-1.13kb upstream SNP) | Forward primer | GACACTCTAAACGATGTGGTGC |
| ­MD016 | *eud-1* promoter region (-1.13kb upstream) | Reverse primer | CCTGCAAGACTGCTAGACTCG |
| MD015 | *eud-1* promoter region (--1.13kb upstream) | Sequencing primer | CCTATATGCACTCGCTTC |
| MD027 | *eud-1* promoter region (-1.97kb upstream SNP) | Forward primer | GGAACCTCACGTAAGGTACTCG |
| MD029 | *eud-1* promoter region (-1.97kb upstream SNP) | Reverse primer | GTCGAAACTTCTAAGAGTCCCG |

| MD030 | *eud-1* promoter region (-1.97kb upstream SNP) | Sequencing primer | CTTCTAAGAGTCCCGTAAG |
| --- | --- | --- | --- |
| MD032 | *eud-1* promoter region (-3.06kb upstream binding motifs ) | Forward primer | GATACAGGCGCTGACGACTG |
| MD033 | *eud-1* promoter region (--3.06kb upstream binding motifs) | Reverse primer | CGCACGGATACACTTCGTCA |
| MD035 | *eud-1* promoter region (-3.06kb upstream binding motifs) | Sequencing primer | TATACTGACTCCAGGCACT |
| MD044 | Highly similar region to the 32-block in *eud-1* first intron | Forward primer | TCACCAAATATCGTGCCTCTTC |
| MD042 | Highly similar region to the 32-block in *eud-1* first intron | Reverse primer | AAGGAGCAGAGCTTGAAGAGGA |
| MD043 | Highly similar region to the 32-block in *eud-1* first intron | Sequencing primer | ATTGACAGTGTCCTCTAAGC |

Swapping experiment were conducted using CRISPR/Cas-9 engineering as described in (8,9). For all swapping experiments a guide RNA complex, which is composed of a 20 base pair target specific oligo CRISPR RNA (crRNA) and a universal trans-activating CRISPR RNA (tracrRNA), with a Cas9 protein, and a single strand DNA repair oligo template were used. This mix was injected into the gonad of a one-day old adult hermaphrodite. The crRNAs were designed upstream to the protospacer adjacent motifs (PAMs) sequences. Repair templates were designed with 40 bases homology arms to each side of the targeted modified swap base, with a total length of approximately 81 bases. Besides the CRISPR/Cas-9 mix to induce swaps in the genome, an *egl-20p*::TurboRFP (PZH009) plasmid was co-injected as a CRISPR/Cas-9 co-injection marker (10). In general, 40 P0 animals were injected, singled out on NGM-OP50 plates and after 4-5 days F1 adults were screened for red-​​fluorescent worms. We singled out F1 animals from the plates with fluorescent progeny. These F1 worms were lysed after F2 progeny collection, and targeted amplicons were amplified, sanger sequenced, and from each heterozygous animal, eight F2 worms were singled out again. After F2 animals had laid eggs, they were lysed, to identify homozygous mutant lines. We used the online pairwise sequence alignment tool EMBOSS Needle with default settings (<https://www.ebi.ac.uk/Tools/psa/emboss_needle/>) to characterize the molecular lesion.

**RNA-seq library preparation and qRT-PCR experiments**

We used synchronized cultures after bleaching to perform RNAseq and qRT-PCR experiments of the *eud-1* gene. Specifically, we used the 36hrs time point that was reported to show highest expression of *eud-1* in the reference strain PS312 (11). Worm pellets were collected in 1.5ml Eppendorf tubes and centrifuged at maximum speed for 2 min. The supernatant was removed and worm pellets were flash frozen in liquid nitrogen and kept at -80°C. For each parental line we prepared two biological replicates. RNA extraction was performed using the Direct-zol Zymo RNA miniprep kit (R2050) following the manufacturer's protocol (Quick protocol) with an elution step was in a final volume of 25µl distilled water. RNA quality was checked using Nanodrop. For Library preparation we used Illumina TruSeq RNA Library Prep Kit, starting with 1ug RNA input. To obtain larger fragments, we increased the timing from 7 to 8 min in the Elute, Prime, Fragment step. We used 12 cycles for PCR enrichment. Samples quantity and quality were checked by Qubit and Bioanalyzer. All samples were diluted to 10nM, and 10ul from each sample were pooled together. The concentration of the pooled library was measured by Qubit and adjusted to 2.5nM before being sequenced on a Hiseq 3000 machine (illumina). For qRT-PCR experiments, we used the iTaq Universal SYBR Green One-Step Kit (#1725150) for measuring normalized expression of *eud-1* in mutant lines and the St parental line RSC011 in relation to the expression in the Eu parental line RSA076. We performed three biological replicates and nine technical replicates. We followed the manufacturer's protocol, while using 20 ng RNA input, and a final concentration of primers 0.25uM as reported in (29). Measuring gene expression was performed on a Roche LightCycler LC480. Program specifications were as follows: reverse transcription reaction 10 min/ 50°C; polymerase activation and DNA denaturation 1 min/ 95°C; denaturation 15 sec/ 95°C; annealing and extension 30 sec/ 60°C; for 45 cycles. Primer sequences used in measuring *eud-1* expression and the reference gene *Ppa-cdc-42* as follows. And relative gene expression was measured by calculating the 2^-delta delta Ct^ relative to RSA076 expression.

| **Primer** | **Sequence 5’----->3’** |
| --- | --- |
| eud-1_qPCR_F | GGCTGGATTCATCACTGGTCGT |
| eud-1_qPCR_R | ATTCCCGTTGCGTAACCTCGT |
| cdc-42_qPCR_F | CTCTCTTATCCACAGACGGAC |
| cdc-42_qPCR_R | GAAGGGAGTGCGTGAGCAGTG |

**Analysis of RNA-seq data**

Raw RNA-seq reads were aligned against the assembly of the *P. pacificus* strain RSB001 (European Nucleotide Archive accession: CAKKKZ010000000) with the tophat2 program (version 2.0.14) using default options (12). Evidence-based gene annotations for the RSB001 genome were created by the PPCAC pipeline (version 1). Specifically, a strain-specific transcriptome assembly (13) and the latest version of the gene annotation for the reference strain PS312 (version: El Paco gene annotation 3) (14) were mapped on the RSB001 assembly, and the longest gene model per 100bp window was chosen as the representative gene model. Estimation of expression levels and differential expression analysis was done by the Cufflinks and Cuffdiff programs (version 2.2.1) using default options (15).

**Statistical analysis**

Mouth-form ratios of the mutants were statistically tested against mouth-form ratios in the parental lines by using the R package betareg to fit beta regression (16). This was performed as mouth-form ratios in some mutants displayed a 0 ratio. Thus, we used this methodology as described in (17). We applied a (y*(n-1)+0.5)/n transformation, where y is the response variable and n is the sample size. Following we applied ANOVA and Post hoc pairwise comparison by using the R package car and lsmeans (18,19).

**Guide RNA and repair templates**

Following is a table with sequences for all guide RNA and repair templates used in this article.

| **Experiment** | **Guide RNA seq 5’--->3’** | **Repair template seq 5’---->3’** |
| --- | --- | --- |
| *nag-2* swap | AGAGAATACGAGGGCTTCAT | ATGCCAAATATTTCGAAATTTCAGAGAATACGAGGGCTTCTTTGGCCACTACTTCATCTGGTGCTTGCTGCAGAACTTTGG |
| *eud-*1 intron swap | GAGAATGAGGAAGTTGATTA | TGAAGAGGAGTCATCTGGAGAATGAGGAAGTTGATTACGGCAGCCGAGGAAATGGAGAAAATAAGTCGGGAGGAAAGATT |
| *eud-1* promoter region (-1.13kb upstream SNP) swap | GCAGACTACGGCTGACAAAT | AGTACGCTGCACAAGTGCGGAAAATGTGCAGACTACGGCTAACAAATAGGAAACCACATCAGTCTCAGCATCGTAACTACC |
| *eud-1* promoter region (-1.97kb upstream SNP) swap | TCGGCGAATTGGTAGCAACT | CCAAGTCTTGCTATCCCTCCGGGGTCGGCGAATTGGTAGCAGCTTTGGTTGCGAAAGGTAAGGCTACCCGAGGAAGCATTG |
| *eud-1* promoter region (-3.06kb upstream binding motifs) | GTTTTTCGATCTCTTGTACA | AAATCTGTGTCACCCCTGCAAACACATCAGATAATGTGCGTGTTTACAATAGATTAGTTTTTCGATCTCTTGTACACGGA |
| Highly similar region to the 32-block in *eud-1* first intron | TACATGAAAAATTAGACTAT | None |

**Population genomic analysis**

Genotypic data from (7) were used to calculate nucleotide diversity in 10-kb windows for different populations. One group was defined by the strains RSC011, RSC012, RSC007, RSC008, and RSC100 which all share the same haplotype at the QTL peak and are all highly St. For the other group, we used all available whole genome sequencing data sets for the NB location in addition to RSC013, RSC172, and RSC009 from CK, all of which share the same haplotype and are highly Eu.

Map generation

The La Reunion island map was generated using the software GeoMapApp (19) after modification from (17).

**Reference**

1. Sieriebriennikov B, Prabh N, Dardiry M, Witte H, Röseler W, Kieninger MR, et al. A Developmental Switch Generating Phenotypic Plasticity Is Part of a Conserved Multi-gene Locus. Cell Rep. 2018 Jun 5;23(10):2835-2843.e4.
2. McGaughran A, Rödelsperger C, Grimm DG, Meyer JM, Moreno E, Morgan K, et al. Genomic Profiles of Diversification and Genotype–Phenotype Association in Island Nematode Lineages. Mol Biol Evol. 2016 May 9;33(9):2257–72.
3. Ragsdale EJ, Müller MR, Rödelsperger C, Sommer RJ. A developmental switch coupled to the evolution of plasticity acts through a sulfatase. Cell. 2013 Nov 7;155(4):922–33.
4. Broman KW. The genomes of recombinant inbred lines. Genetics. 2005 Feb;169(2):1133–46.
5. Li H, Durbin R. Fast and accurate short read alignment with Burrows–Wheeler transform. Bioinformatics. 2009 May 18;25(14):1754–60.
6. Lenuzzi M, Witte H, Riebesell M, Rödelsperger C, Hong RL, Sommer RJ. Influence of environmental temperature on mouth-form plasticity in Pristionchus pacificus acts through daf-11-dependent cGMP signaling. J Exp Zool B Mol Dev Evol [Internet]. 2021 Aug 11;n/a(n/a). Available from: https://doi.org/10.1002/jez.b.23094
7. Rödelsperger C, Meyer JM, Prabh N, Lanz C, Bemm F, Sommer RJ. Single-Molecule Sequencing Reveals the Chromosome-Scale Genomic Architecture of the Nematode Model Organism Pristionchus pacificus. Cell Rep. 2017 Oct 17;21(3):834–44.
8. Rae R, Witte H, Rödelsperger C, Sommer RJ. The importance of being regular: Caenorhabditis elegans and Pristionchus pacificus defecation mutants are hypersusceptible to bacterial pathogens. Int J Parasitol. 2012 Jul;42(8):747–53.
9. Witte H, Moreno E, Rödelsperger C, Kim J, Kim J-S, Streit A, et al. Gene inactivation using the CRISPR/Cas9 system in the nematode Pristionchus pacificus. Dev Genes Evol. 2015 Jan;225(1):55–62.
10. Lightfoot JW, Wilecki M, Rödelsperger C, Moreno E, Susoy V, Witte H, et al. Small peptide-mediated self-recognition prevents cannibalism in predatory nematodes. Science. 2019 Apr 5;364(6435):86–9.
11. Han Z, Lo W-S, Lightfoot JW, Witte H, Sun S, Sommer RJ. Improving Transgenesis Efficiency and CRISPR-Associated Tools Through Codon Optimization and Native Intron Addition in Pristionchus Nematodes. Genetics. 2020 Dec;216(4):947–56.
12. Werner MS, Sieriebriennikov B, Loschko T, Namdeo S, Lenuzzi M, Dardiry M, et al. Environmental influence on Pristionchus pacificus mouth form through different culture methods. Sci Rep. 2017 Aug 3;7(1):7207.
13. Rödelsperger C. The community-curated Pristionchus pacificus genome facilitates automated gene annotation improvement in related nematodes. BMC Genomics. 2021 Mar 25;22(1):216.
14. Rödelsperger C, Röseler W, Prabh N, Yoshida K, Weiler C, Herrmann M, et al. Phylotranscriptomics of Pristionchus Nematodes Reveals Parallel Gene Loss in Six Hermaphroditic Lineages. Curr Biol. 2018 Oct 8;28(19):3123-3127.e5.
15. Athanasouli M, Witte H, Weiler C, Loschko T, Eberhardt G, Sommer RJ, et al. Comparative genomics and community curation further improve gene annotations in the nematode Pristionchus pacificus. BMC Genomics. 2020 Oct 12;21(1):708.
16. Trapnell C, Hendrickson DG, Sauvageau M, Goff L, Rinn JL, Pachter L. Differential analysis of gene regulation at transcript resolution with RNA-seq. Nat Biotechnol. 2013 Jan;31(1):46–53.
17. Cribari-Neto F, Zeileis A. Beta Regression in R. J Stat Softw [Internet]. 2010 Apr 5 [cited 2021 Dec 18];034(i02). Available from: https://EconPapers.repec.org/RePEc:jss:jstsof:v:034:i02
18. Smithson M, Verkuilen J. A better lemon squeezer? Maximum-likelihood regression with beta-distributed dependent variables. Psychol Methods. 2006 Mar;11(1):54–71.
19. Fox J, Weisberg S, Adler D, Bates D, Baud-Bovy G, Ellison S, et al. Package ‘car.’ Vienna: R Foundation for Statistical Computing. 2012;16.
