# Supplementary figures and images for: Divergent combinations of cis-regulatory elements control the evolution of phenotypic plasticity
Source: PLoS Biol. 2023 Aug 17;21(8):e3002270. doi: 10.1371/journal.pbio.3002270 (PMC10464979; doi:10.1371/journal.pbio.3002270)

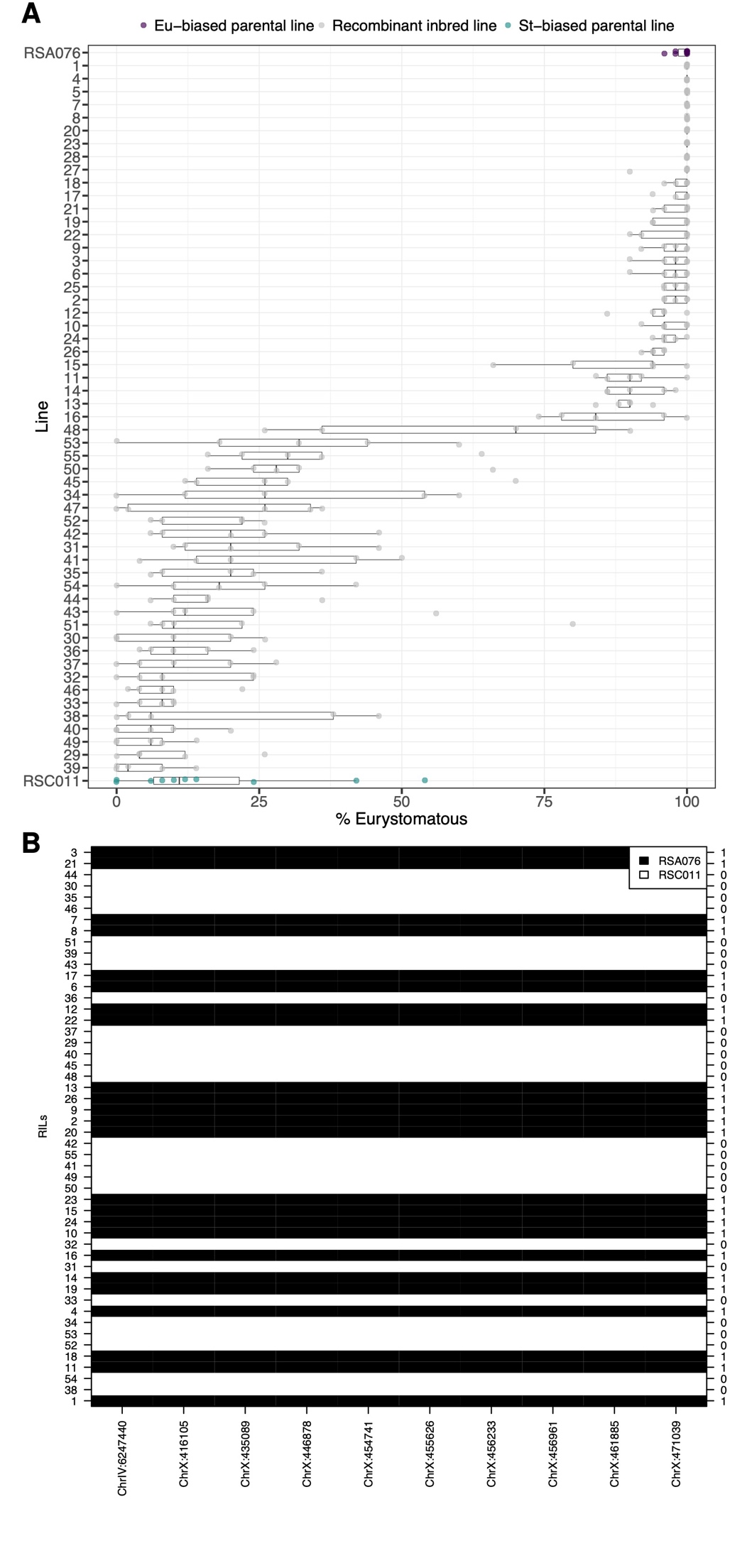

Supplement: S1 Fig — (A) Mouth-form ratios of a subset of the RILs representative of the full range of phenotypes. (B) Highly significant markers of ChrV and ChrIV display similar inheritance patterns as ChrX markers. Theses similarities indicate genetic linkage between the markers, which, in turn, implies that all these markers must be on the same chromosome. The x-axis displays the 55 RILs, and the y-axis indicates chromosomal markers. For detailed information, see S3 Data. (DOCX) [file pbio.3002270.s002.docx]

**
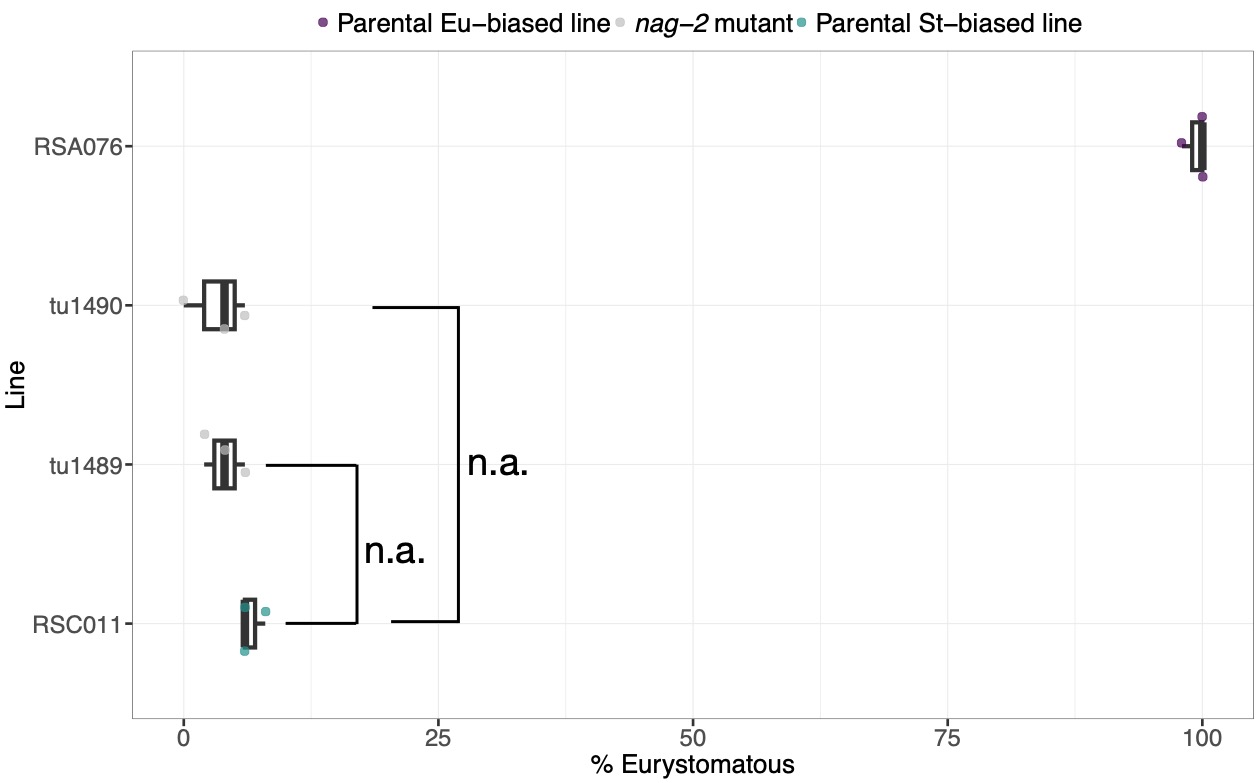
**

Supplement: S2 Fig — Mutant lines tu1489 and tu1490 were generated by introducing the RSA076 variant in the RSC011 background, i.e., swapping (A) with (T). Mouth-form score was counted for 3 replicates each line. Statistical analysis shows no significant difference between the mutant lines and RSC011. For detailed information, see S4 Data. (DOCX) [file pbio.3002270.s003.docx]

**
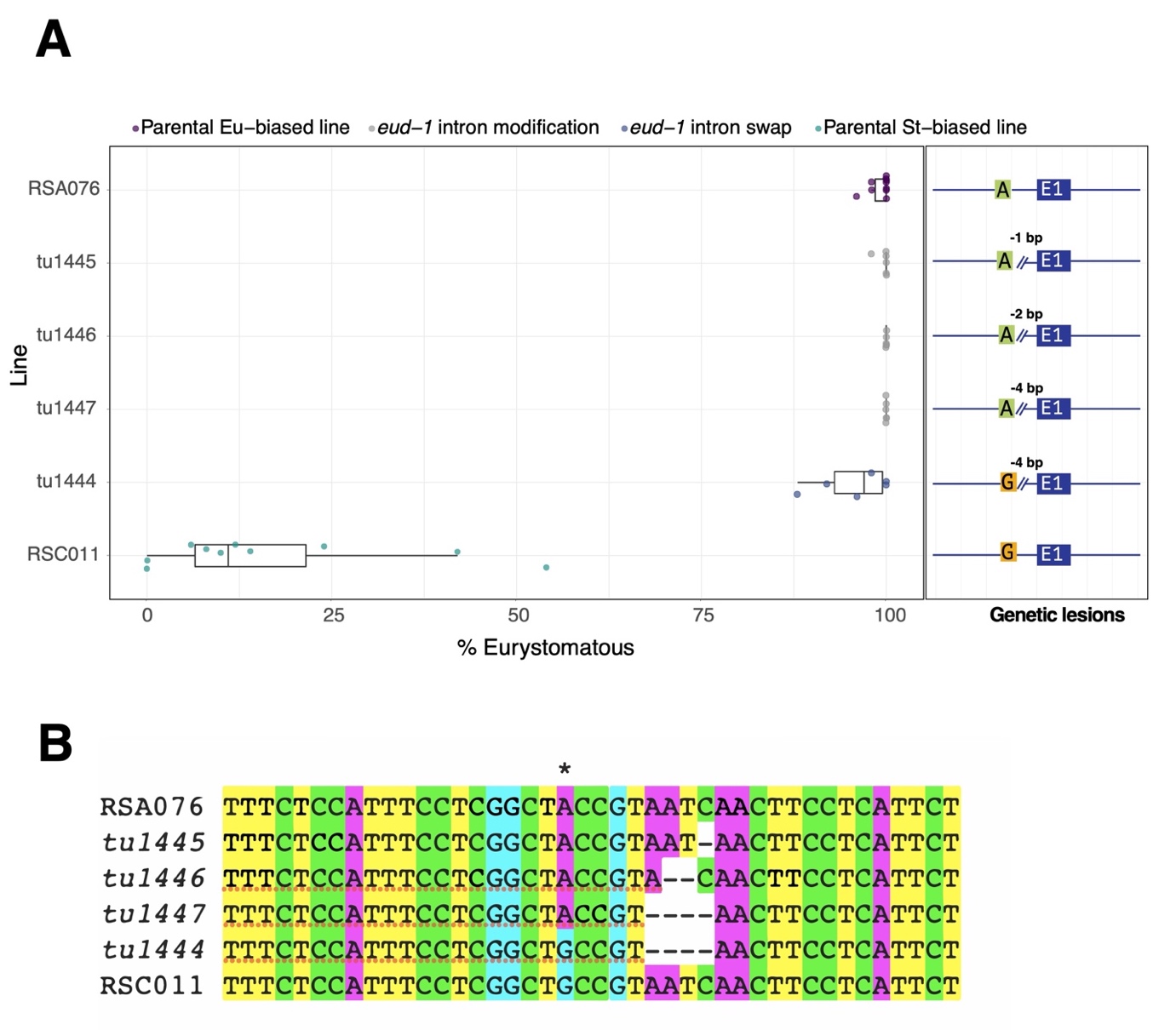
**

Supplement: S5 Fig — (A) Mutants with downstream deletion to the intronic swap variant; tu1445, tu1446, tu1444 display weaker mouth-form change than the mutant with the targeted swap; tu1444. Both tu1447 and tu1444 harbor the same 4-bp deletion. For detailed information, see S6 Data. (B) Sequence alignment of the mutants. The nucleotide under the star represents the targeted swapped nucleotide. Dashes represent deletions. (DOCX) [file pbio.3002270.s006.docx]

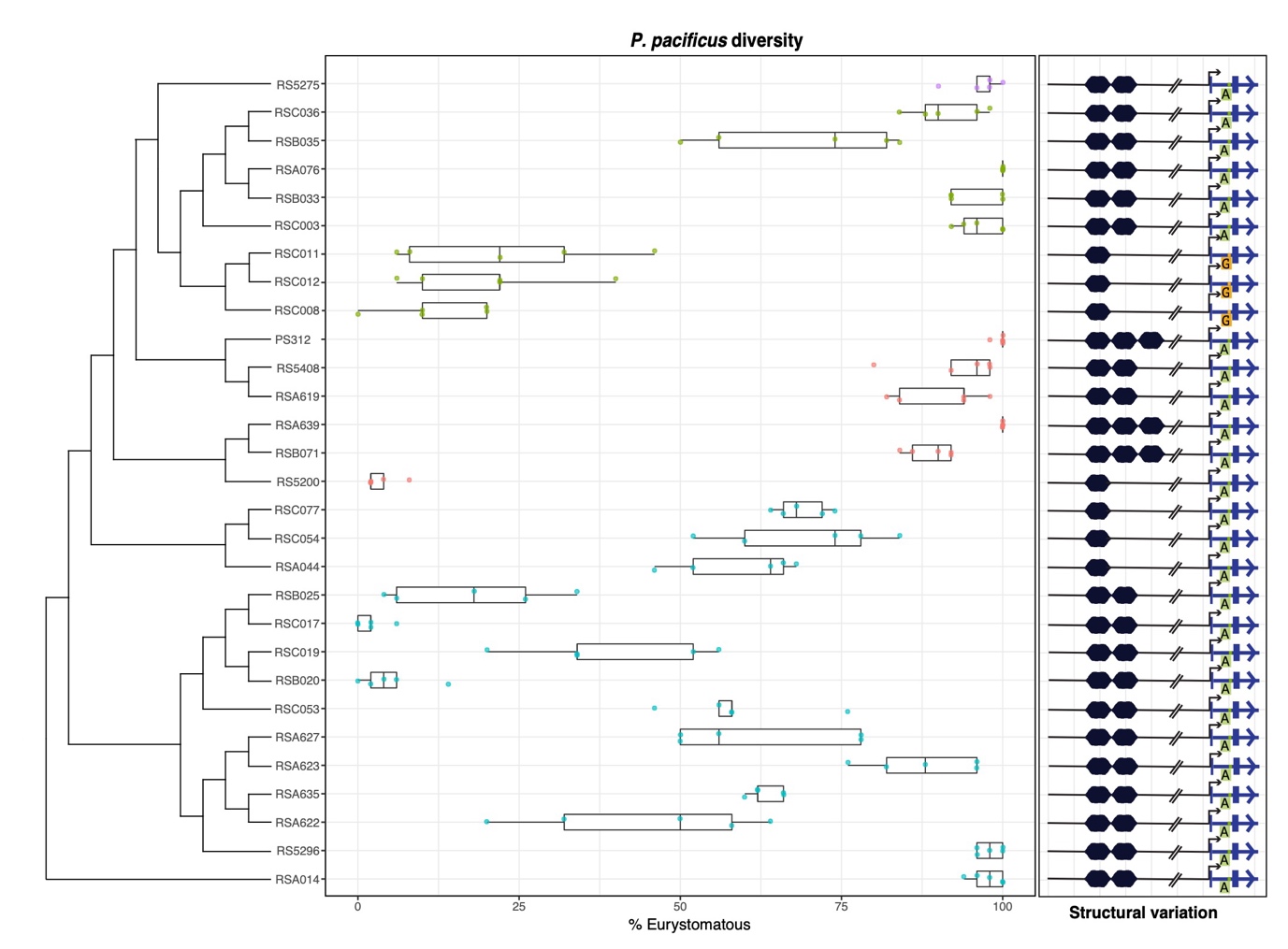

Supplement: S6 Fig — Several strains including the wild-type PS312 from California have 3 copies of the transcription factor binding site and contain the intronic “A” polymorphism similar to RSA076. Strain color code: green: clade B; orange: clade A; blue: clade C and light purple: outgroup. For detailed information, see S7 Data. (DOCX) [file pbio.3002270.s007.docx]

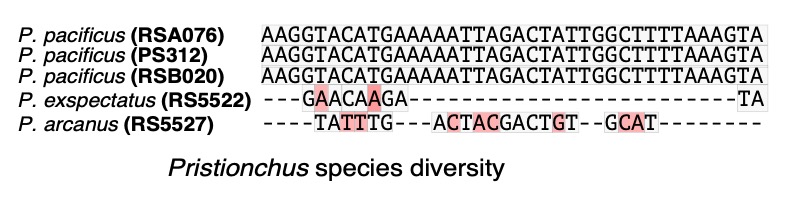

Supplement: S7 Fig — Intronic sequence highly similar to the 32-nucleotide block in the eud-1 promoter, (+72 bp), display sequence conservation across P. pacificus clades. RSA076 represents clade B, PS312 represents clade A, and RSB020 represents clade C. The same intronic sequence show divergence when compared to the most closely related Pristionchus species (P. exspectatus and P. arcanus). Dashes represent deletions, and red boxes represent nucleotide variation. (DOCX) [file pbio.3002270.s008.docx]

**
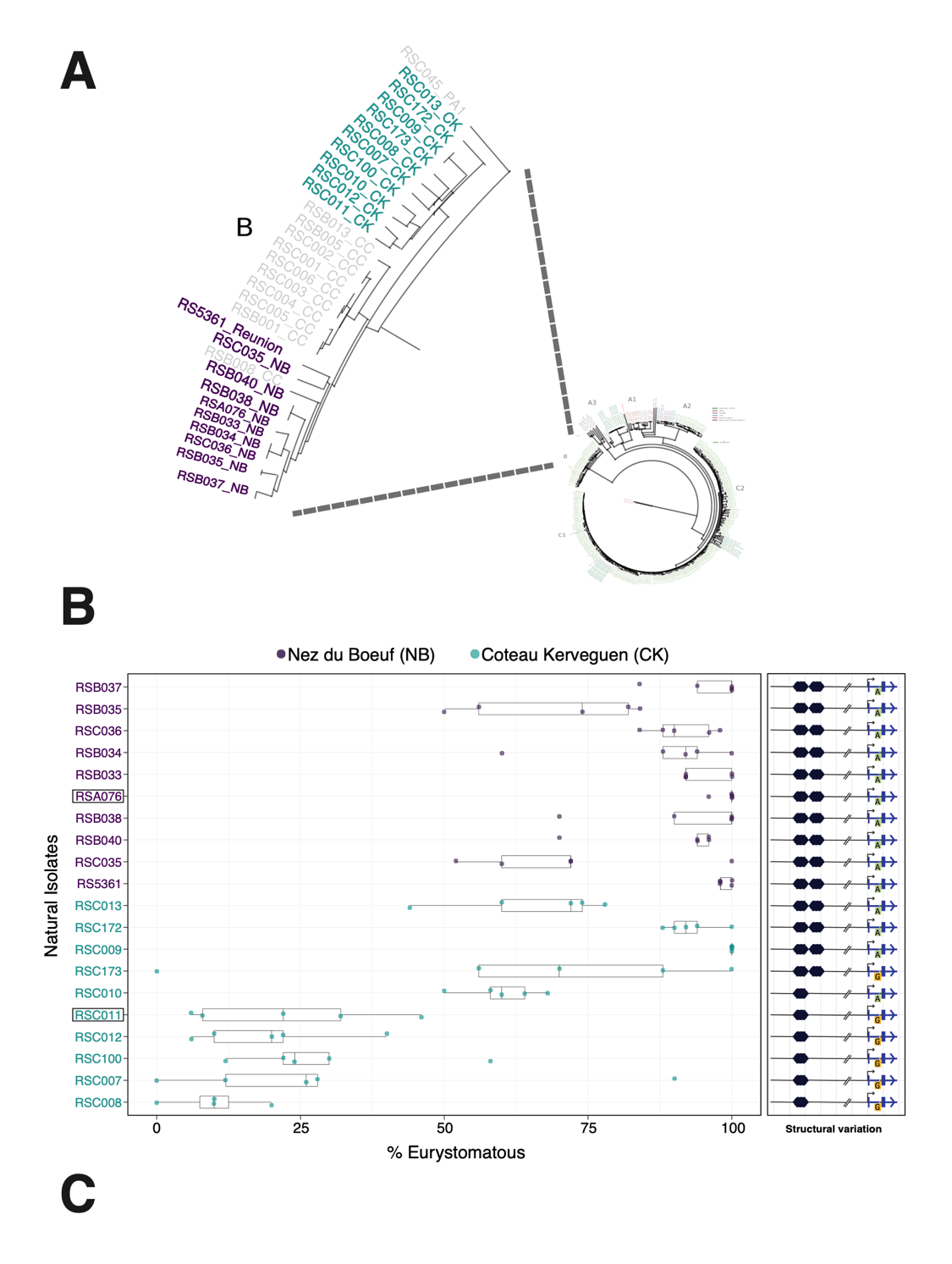
**
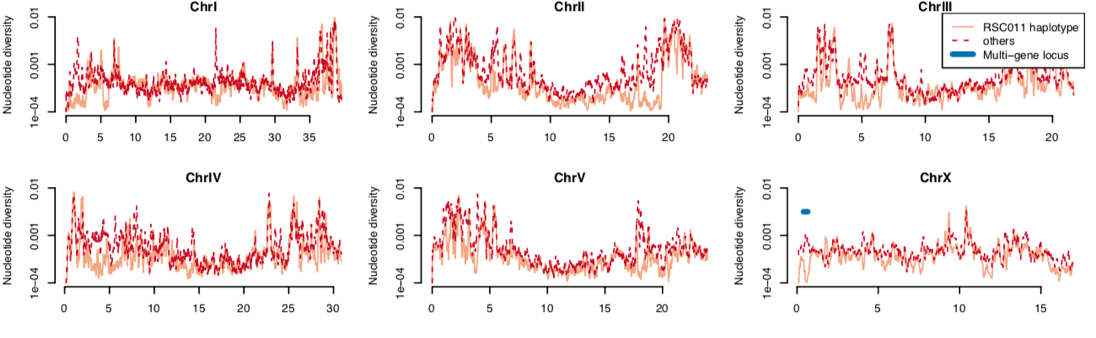

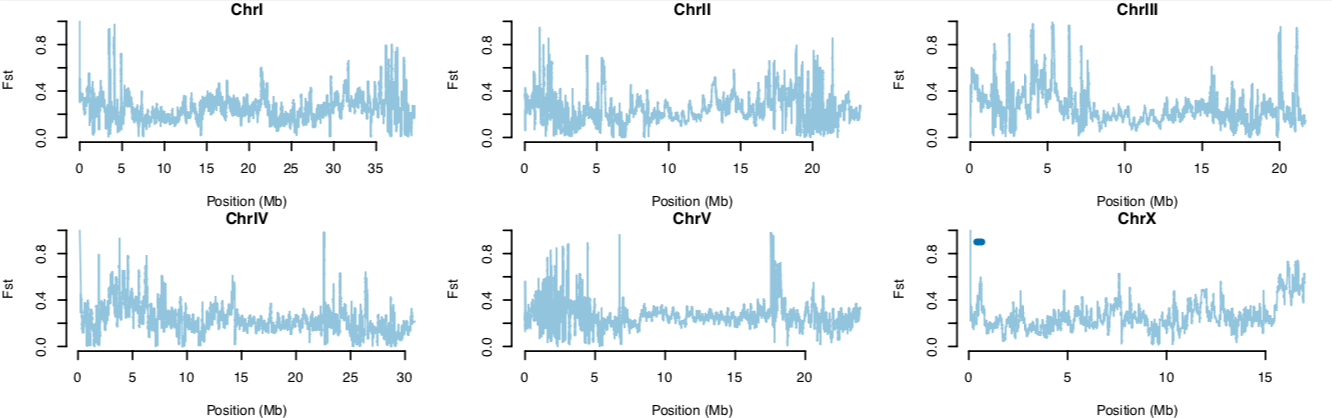

Supplement: S8 Fig — (A) Extended phylogeny of clade B strains of P. pacificus. Note that only 10 strains are available from CK, one of the most remote places on La Réunion Island. Only a subset of the many strains of NB and CC are shown. (B) Mouth-form divergence and natural variation at the eud-1 locus of all 10 CK-derived and 10 selected NB-derived strains. Only 4 CK strains share the RSC011 haplotype, whereas the majority of the others strains have the RSA076 haplotype consistent with RSC011 representing the derived character. (C) Nucleotide diversity and Fst data of the 20 strains from CK and NB based on population-scale whole-genome sequencing. CK strains with the RSC011 haplotype were compared to NB and CK strains with the RSA076 haplotype. For detailed information, see S8 Data. (DOCX) [file pbio.3002270.s009.docx]

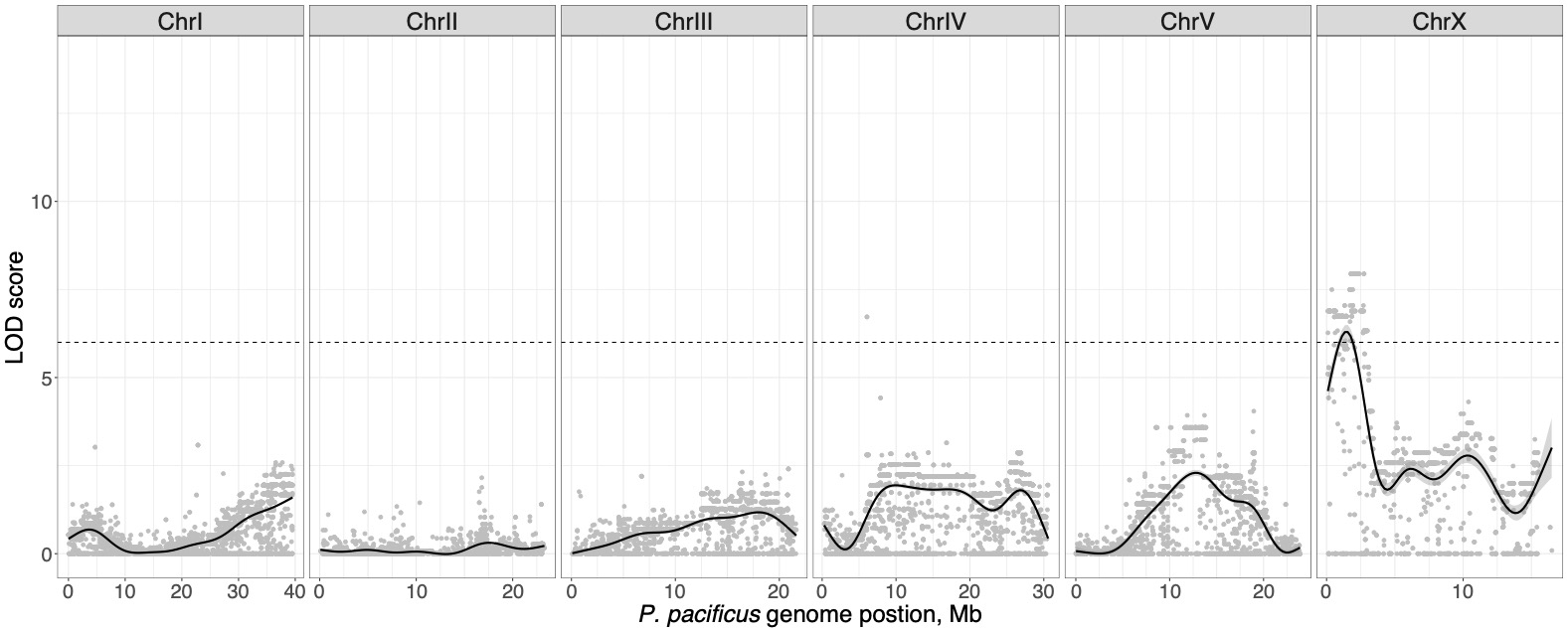

Supplement: S9 Fig — QTL analysis of 94 RILs reveals a single peak of around 900 kb at the left end of the X chromosome. For detailed information, see S9 Data. (DOCX) [file pbio.3002270.s010.docx]
