# Supplementary material for: Divergent combinations of cis-regulatory elements control the evolution of phenotypic plasticity
Source: PLoS Biol. 2023 Aug 17;21(8):e3002270. doi: 10.1371/journal.pbio.3002270 (PMC10464979; doi:10.1371/journal.pbio.3002270)
Supplement: S1 Table — N = 150, 3 replicates (n = 50) for all lines. % Eu, percent eurystomatous animals; n.a., not applicable. Genomic position in relation to RSB001 reference genome. (DOCX) [file pbio.3002270.s011.docx]

| **Genotype** | **Background** | **Molecular lesion** | **Genomic position (RSB001)** | **Average %Eu** |
| --- | --- | --- | --- | --- |
| RSA076 | n.a. | n.a. | n.a. | 99.3% |
| *tu1489* | RSC011 | Swap (A>T) exon 10 | Scaffold129: 24,87,85 | 4% |
| *tu1490* | RSC011 | Swap (A>T) exon 10 | Scaffold129: 24,87,85 | 3.3% |
| RSC011 | n.a. | n.a. | n.a. | 6.6% |
