# Supplementary material for: Divergent combinations of cis-regulatory elements control the evolution of phenotypic plasticity
Source: PLoS Biol. 2023 Aug 17;21(8):e3002270. doi: 10.1371/journal.pbio.3002270 (PMC10464979; doi:10.1371/journal.pbio.3002270)
Supplement: S2 Table — Highest number of intergenic SNPs was detected in the intergenic region between eud-1 and sul.2.2.1. (DOCX) [file pbio.3002270.s012.docx]

| **Region** | **Genomic position (RSB001)** | **Number of variants** | **Gene identifier** |
| --- | --- | --- | --- |
| *nag-2* | Scaffold129:  245,646-251,539 | 5 | RSB001000005737 |
| Intergenic region between *sul.2.2.1* and *nag-2* | Scaffold129:  251,540-254,138 | 4 | n.a. |
| *­­­sul.2.2.1* | Scaffold129:  254,139-258,900 | 6 | RSB001000006991 |
| Intergenic region between *eud-1* and *sul.2.2.1* | Scaffold129:  258,901-264,675 | 7 | n.a. |
| *eud-1* | Scaffold129:  264,676-269,511 | 11 | RSB001000004808 |
| Intergenic region between *nag-1* and *eud-1* | Scaffold129:  269,512-270,428 | 2 | n.a. |
| *nag-1* | Scaffold129:  270,427-275,873 | 6 | RSB001000006459 |
