# Supplementary material for: Divergent combinations of cis-regulatory elements control the evolution of phenotypic plasticity
Source: PLoS Biol. 2023 Aug 17;21(8):e3002270. doi: 10.1371/journal.pbio.3002270 (PMC10464979; doi:10.1371/journal.pbio.3002270)
Supplement: S4 Table — N = 300, 6 replicates (n = 50) for mutants and N = 500, 10 replicates (n = 50) for parental lines; % Eu, percent eurystomatous animals; n.a., not applicable. Genomic position in relation to RSB001 reference genome. (DOCX) [file pbio.3002270.s014.docx]

| **Genotype** | **Background** | **Molecular lesion** | **Genomic position (RSB001)** | **Average %Eu** |
| --- | --- | --- | --- | --- |
| RSA076 | n.a. | n.a. | n.a. | 99.2% |
| *tu1444* | RSA076 | *eud-1*first intron variant (+182b)  Swap (A>G) + 4 bp deletion | Scaffold 129:  26,48,97 | 95.6% |
| *tu1590* | *tu1504* | Intergenic variant (-3.06 kb)  45 bp deleted including one FBS  + 36 bp insertion | Scaffold 129:  26,16,40-26,16,70 | 90.6% |
| *tu1621* | *tu1590* | Intergenic variant (-3.06 kb)  36 bp deleted including one FBS | Scaffold 129:  26,16,40-26,16,70 | 81% |
| *tu1866* | *tu1590* | Intergenic variant (-3.06 kb)  74 bp deleted including the two FBS + 6 bp insertions | Scaffold 129:  26,16,02-26,16,70 | 74% |
| *tu1867* | *tu1590* | Intergenic variant (-3.06 kb)  64 bp deleted including the two FBS + 6 bp insertions | Scaffold 129:  26,16,02-26,16,70 | 73% |
| *tu1868* | *tu1621* | *eud-1*first intron variant (+182b)  Swap (A>G) + 4 bp deletion | Scaffold 129:  26,48,97 | 37% |
| *tu1869* | *tu1621* | *eud-1*first intron variant (+182b)  Swap (A>G) + 1 bp substitution | Scaffold 129:  26,48,97 | 51.6% |
| *tu1870* | *tu1867* | *eud-1*first intron variant (+182b)  Swap (A>G) + 23 bp  insertion | Scaffold 129:  26,48,97 | 9% |
| *tu1871* | *tu1867* | *eud-1*first intron variant (+182b)  Swap (A>G) +10 bp  Insertion + 6 bp deletion | Scaffold 129:  26,48,97 | 15.3% |
| RSC011 | n.a. | n.a. | n.a. | 17% |
