# Supplementary material for: Divergent combinations of cis-regulatory elements control the evolution of phenotypic plasticity
Source: PLoS Biol. 2023 Aug 17;21(8):e3002270. doi: 10.1371/journal.pbio.3002270 (PMC10464979; doi:10.1371/journal.pbio.3002270)
Supplement: S5 Table — N = 250, 5 replicates (n = 50) for mutants and N = 500, 10 replicates (n = 50) for parental lines; % Eu, percent eurystomatous animals; n.a., not applicable. Genomic position in relation to RSB001 reference genome. (DOCX) [file pbio.3002270.s015.docx]

| **Genotype** | **Background** | **Molecular lesion** | **Genomic position (RSB001)** | **Average %Eu** |
| --- | --- | --- | --- | --- |
| RSA076 | n.a. | n.a. | n.a. | 99.2% |
| *tu1901* | RSA076 | 2 bp deletion + 2 bp substitution | Scaffold 129:  26,47,87-26,48,24 | 100% |
| *tu1902* | RSA076 | 5 bp deletion | Scaffold 129:  26,47,87-26,48,24 | 99.6% |
| *tu1903* | RSA076 | 6 bp deletion | Scaffold 129:  26,47,87-26,48,24 | 86% |
| *tu1904* | RSA076 | 4 bp insertion + 1 bp substitution | Scaffold 129:  26,47,87-26,48,24 | 32.2% |
| *tu1905* | RSA076 | 31 bp deletion | Scaffold 129:  26,47,87-26,48,24 | 0% |
| *tu1906* | RSA076 | 31 bp deletion | Scaffold 129:  26,47,87-26,48,24 | 0% |
| RSC011 | n.a. | n.a. | n.a. | 17% |
