# Supplementary material for: Divergent combinations of cis-regulatory elements control the evolution of phenotypic plasticity
Source: PLoS Biol. 2023 Aug 17;21(8):e3002270. doi: 10.1371/journal.pbio.3002270 (PMC10464979; doi:10.1371/journal.pbio.3002270)
Supplement: Supplementary file 8 [file pbio.3002270.s004.docx]

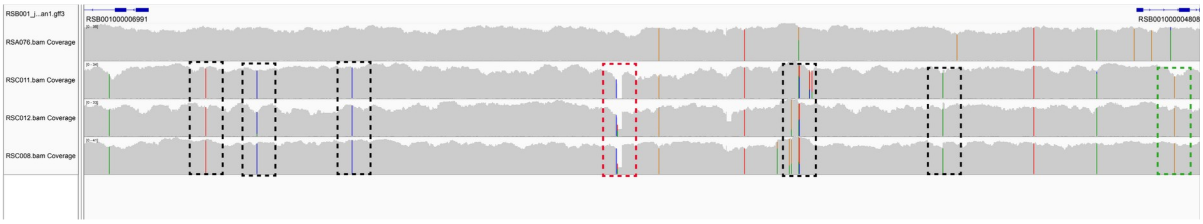

RSB001_j...an1.gff3
RSB001000006991
RSB001000004808
RSA076.bam Coverage
[0 - 36]
RSC011.bam Coverage
[0 - 34]
RSC012.bam Coverage
[0 - 33]
RSC008.bam Coverage
[0 - 41]
