# Supplementary material for: Divergent combinations of cis-regulatory elements control the evolution of phenotypic plasticity
Source: PLoS Biol. 2023 Aug 17;21(8):e3002270. doi: 10.1371/journal.pbio.3002270 (PMC10464979; doi:10.1371/journal.pbio.3002270)
Supplement: Supplementary file 9 [file pbio.3002270.s005.docx]

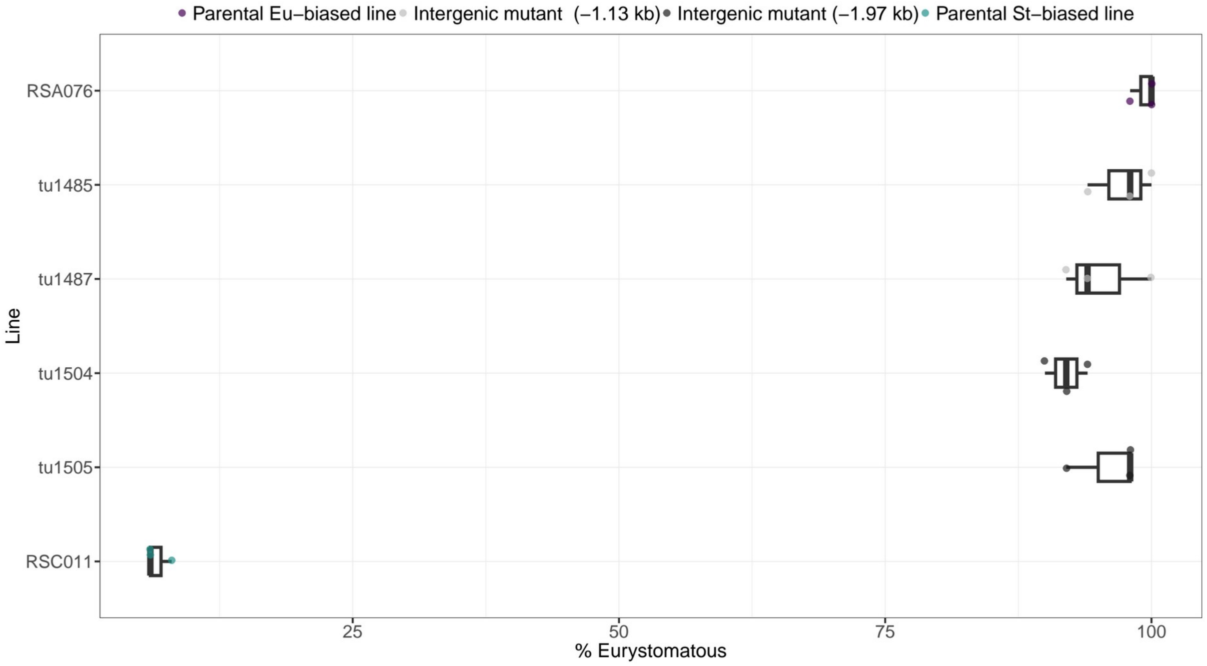
Parental Eu−biased line
Intergenic mutant (−1.13 kb)
Intergenic mutant (−1.97 kb)
Parental St−biased line
RSA076
tu1485
tu1487
tu1504
tu1505
RSC011
Line
25
50
75
100
% Eurystomatous
