# Supplementary material for: Divergent combinations of cis-regulatory elements control the evolution of phenotypic plasticity
Source: PLoS Biol. 2023 Aug 17;21(8):e3002270. doi: 10.1371/journal.pbio.3002270 (PMC10464979; doi:10.1371/journal.pbio.3002270)
Supplement: Attachment — Submitted filename: Point-by-point Response_V1.docx [file pbio.3002270.s025.docx]

Dear Dr Alvarez-Garcia,

Thank you for your positive evaluation of our manuscript. We have now revised the manuscript according to the reviewer´s suggestions. Please find our point-by-point response below


**DATA POLICY: IMPORTANT - PLEASE READ**

Response: We now provide the individual numerical values that underlie the data displayed in the figure panels.


**Reviewer 1:**

Response: We thank the reviewer for the very positive evaluation of our manuscript.


**Reviewer 2:**

Page 3, par. 2:
Figure 1E's F12 diagrams don't reflect the homozygosity expected from selfing for this long.

Response: We thank the reviewer for catching this oversight. We have now corrected Fig. 1E to indicate the expected homozygosity.

Page 4, bottom par:
It is interesting that simultaneous deletion of one copy of the upstream repeated element and conversion of the eud-1 intron 1 SNP in RSA076, which largely converts the QTL to the RSC011 genotype, is not sufficient to perfectly mimic the RSC011 phenotype of 20% Eu formation. Only when both repeats are deleted do we get to the RSC011 level. This should be discussed at some point. One also wonders whether, in such a background, other QTL might be identifiable that also contribute to the all-Eu state of RSC076.

Response: We have added a short discussion of this result on page 5. We agree that other QTLs that contribute to the all-Eu phenotype of RSA076 could in theory be identifiable in this background. However, this would have required the accumulation of CRISPR-induced ‘mutations’ and could easily have resulted in another 1-2 year´s work.

Page 6, par. 1 and Pages 14-15 (Figure 3 and associated text):
Regarding a possible selective sweep of the RSC011 QTL allele: This is one possibility, but I am skeptical. It would seem that once we specify any specific, recently evolved haplotype among a larger population set, we expect the diversity to fall compared to the whole set. Further, that the RSC011 haplotype is not fixed even in the Clade B worms that live in the spatially confined Nez de Boeuf A. godefroyi-associated habitat in which both RSC011 and RSA076 live actually suggests the opposite: a tendency to form the Eu mouthform is not under strong selection, and thus a recently evolved, mild loss-of-function allele in eud-1 is basically neutral. Overall, a claim or suggestion of a selective sweep seems premature.

Result: We agree with the reviewer and have removed the suggestion/statement about a potential selective sweep from the manuscript. Also, we made it clear that we can currently not distinguish between neutrality and natural selection shaping the evolution of this locus.

Pages 12-13:
Figure 2 needs some help to be easily interpreted. A few specific issues first:
* In panel B, the label "P. pacificus genome scale, Mb" is not helpful. It appears to be copied over from the bottom of panel A, but here it is clear kilobases (Kb), not Mb.
* The numbered scale on the read coverage plot has some digits out of order (from 3-4).
The biggest issue: I struggled to reconcile the lower part of panel B with panel C, but finally figured it out: In panel B the first, conserved repeat copy in 076 is in gray at the left, while the second is part of the strain-specific yellow sequence. It would be more clear to get rid of panel C, and modify panel B to better indicate the repeated part. For example, an arrow indicating the extent of each copy over the alignment would be very clear. The end result will have the same information, but take less space and be easier to comprehend.

Response: We thank the reviewer again for catching our small errors.

Also, we have followed the advice and have removed former panel ‘Fig. 2C'.

In summary, we have addressed all issues raised by the reviewer.

Thank you very much for considering this revised manuscript.

On behalf of all co-authors

Ralf J. Sommer
